# Performance of automatic capture confirmation algorithm in a large cohort of pacemaker patients with left bundle branch area pacing

**DOI:** 10.1007/s10840-025-02229-y

**Published:** 2026-02-23

**Authors:** Matthew A. Bernabei, Sandeep Bansal, R. Ward Pulliam, Fady Dawoud, Wenwen Li, Leyla Sabet, Kyungmoo Ryu, Luke McSpadden, Jeffrey Arkles

**Affiliations:** 1 Penn Medicine - Lancaster General Health, Lancaster, PA USA; 2 Abbott, Sylmar, CA USA

**Keywords:** Left bundle branch area pacing, Conduction system pacing, Capture threshold management, AutoCapture, Cardiac implantable devices, Remote monitoring

## Abstract

**Background:**

Automatic capture confirmation algorithms regularly evaluate pacing capture threshold (PCT) and adjust energy output to deliver a tailored safety margin over the PCT. Abbott AutoCapture™ Algorithm provides beat-by-beat capture confirmation and delivers a high-output backup safety pulse in the event of non-capture. Our objective was to evaluate the longitudinal performance and stability of the Abbott AutoCapture™ algorithm in patients with LBBAP.

**Methods:**

De-identified remote device data were retrospectively analyzed from consecutive patients in our hospital who received AutoCapture enabled Abbott pacemakers with LBBAP from June 2021 to August 2023. Device stored AutoCapture PCT measurements were then evaluated incrementally over an approximate 2-year period, to evaluate longer-term trends and performance, and also compared with the in-clinic manual PCT.

**Results:**

A total of 619 patients with either single chamber (*n* = 89) or dual-chamber Abbott devices (*n* = 530) were identified. AutoCapture and manually measured PCTs in-clinic were within 0.25 V in 600/615 (97.6%) patients, with average PCTs of 0.76 V ± 0.28 and 0.80 V ± 0.26 respectively. At 1, 3, 6, 12, and 24-month remote follow-up, average AutoCapture PCTs were 0.67 V ± 0.29 (*n* = 594), 0.66 V ± 0.25 (*n* = 560), 0.71 V ± 0.29 (*n* = 543), 0.77 V ± 0.29 (*n* = 447) and 0.81 V ± 0.28 (*n* = 112), respectively. AutoCapture was found to be effective in assessing PCT and was activated in the majority of patients (619/644, 97%) with no clinical complications related to its usage.

**Conclusion:**

The AutoCapture algorithm measured accurate PCTs compared to manual in-clinic and showed stable trend during follow-up of 2 years in patients with LBBAP.

**Supplementary Information:**

The online version contains supplementary material available at 10.1007/s10840-025-02229-y.

## Introduction

Cardiac implantable electronic devices equipped with automatic capture optimization and confirmation algorithms dynamically adapt pacing output to maintain effective cardiac capture and prolong device longevity thereby delaying generator replacement. In combination with remote interrogation, this allows convenient monitoring of device function and lead electrical parameters thereby enhancing patient safety [9]. More specifically, AutoCapture automatically determines the PCT and delivers pacing with a 0.25 V safety margin while continually verifying beat-by-beat capture by examining evoked ventricular response (EVR) and providing back-up pacing at 5 V when lack of capture is determined.

Conduction system pacing (CSP), now most commonly achieved through LBBAP, has emerged over the last several years as the predominant alternative to traditional right ventricular pacing (RVP) [1–3]. It mitigates many of the known associated issues inherent to conventional RVP and therefore continues to gain increasing acceptance and clinical use. LBBAP usually allows for standard programming with pacing parameters similar to RVP, typically with a low output pacing amplitude, and favorable sensing parameters [4–7]. AutoCapture™ has been well validated for RVP [8], however, there is limited published data on the algorithm’s performance in a large LBBAP population. Considering the unique anatomical and electrophysiological properties of the LBBA, pacing output dependent morphological changes of evoked response seen with LBBAP combined with the many potential superimposed variables when placing a pacing lead in this position, we sought to better understand, characterize and validate the performance of AutoCapture for this burgeoning and increasingly utilized form of pacing.

In our study, we evaluated the initial and then medium-term (up to 2 years) performance (through remote monitoring) of the Abbott (Chicago, IL, USA) AutoCapture algorithm in patients implanted with LBBAP.

## Methods

### AutoCapture algorithm

AutoCapture algorithm initiates PCT search every 8 hours or sooner when loss of capture is suspected. The threshold search occurs over a series of cardiac cycles using a programmable shortened AV delay (50 ms for paced and 25 ms for sensed atrial beats) at a fixed 0.5 ms pulse width. The PCT is determined as the first threshold with 2 consecutive confirmed captured beats after decremental then incremental output. A high-output 5 V backup pacing pulse is delivered within 100 ms in cases of loss of capture to ensure beat-by-beat capture. If PCT is not found during the threshold search, the device switches to high-output pacing mode and restarts a threshold search after 128 beats. A fusion avoidance mechanism is used during beat-by-beat verification to extend AV delay by up to 100 ms to promote intrinsic conduction thereby minimizing ineffective double pacing. The EVR channel in pacemakers can either be programmed to unipolar RVtip-to-Can with bipolar pacing (nominal setting) or bipolar RVtip-to-RVring with unipolar pacing. The EVR waveform in an interval subsequent to pacing is compared to an EVR sensitivity level determined during in-clinic setup test. The EVR’s waveform dynamic range and sensitivity level are automatically selected during the setup test to ensure proper sensing of the EVR waveform with a safety margin over lead polarization artifacts and at the same time with an adequate safety margin to reliably differentiate EVR capture from non-capture beats. The paced depolarization integral (PDI) method based on area-under-curve is nominally used with bipolar pacing [8].

### Study design and analysis

We performed a retrospective non-randomized single-center review of our patients where LBBAP is routinely performed. De-identified Merlin.Net™ transmissions were obtained in all LBBAP patients receiving AutoCapture capable pacemakers between June 2021 and August 2023. LBBAP was performed with either a catheter-driven lumen-less fixed helix lead or a stylet-driven retractable helix lead.

The LBBAP implant procedure was performed per established protocol at Penn Medicine—Lancaster General Hospital. Targeting of the left-bundle branch in the ventricular septum and LBBAP success were based on published criteria [10, 11]. AutoCapture activation was attempted at implant using the programmer (Merlin Patient Care system) automated setup test with same workflow as traditional RVA with bipolar pacing configuration as first option. Manual and AutoCapture decrement tests were performed to determine PCT. Test data including EGM waveforms automatically stored in the pacemaker were used for retrospective analysis to adjudicate capture threshold levels. Remote transmission data were analyzed during the first device programming session and longitudinally over 1, 3, 6, 12, and 24-month follow-up durations. In addition, electrical characteristics of lead parameters (PCT, sensing amplitude and pacing impedance) were aggregated longitudinally. Study design was conceived by MB and WL. Data analysis was performed by MB and FD. Manuscript draft was performed by MB and JA. Manuscript revision was performed by all authors.

### Statistical analysis

All statistical analyses were performed using Matlab R2020a. Categorical data were expressed as number and percentage. Quantitative variables were expressed as mean and standard deviation. Comparison for equivalence of manual and PCT was performed using two-sided t-test method, setting a 95% confidence interval and considering a difference in PCT < 0.25 V as equivalent from a clinical relevance standpoint.

## Results

### Baseline characteristics

Among a total of 644 patients implanted with a pacemaker and LBBAP lead between June 2021 and August 2023 for bradycardia indication, 619 patients (age 80 ± 9 years) had AutoCapture activated and adequate data for analysis (3 had < 30 days follow-up, 20 didn’t have the AutoCapture setup test performed, 2 had setup test didn’t pass, more details provided in Supplementary Section 1.0).

Among the 619 patients, 89 (14%) received an Abbott Assurity MRI single-chamber pacemaker (model 1272) and 530 (86%) received an Abbott Assurity MRI dual-chamber pacemaker (model 2272). LBBAP was performed with a catheter-driven lumen-less fixed helix lead (SelectSecure 3830, Medtronic) in 630 patients and a stylet-driven retractable helix lead (Tendril 2088, Abbott) in 14 patients (details on characteristics of lead subgroups available in Supplementary Section 2.0). Programmed sensed AV delay was 151 ± 23 ms and paced AV delay was 184 ± 20 ms (main results in Table 1).

**Table 1:** AutoCapture Algorithm PCT agreement with manual measurement and electrical performance over 2-year follow-up

	*N*	Mean	Std Dev
Manual PCT (V)	615	0.8	0.26
Automatic AutoCapture PCT (V)	615	0.76	0.28
	**N (N < 2 V)**	**Mean**	**Std Dev**
AutoCapture PCT 1-month (V)	594 (588)	0.67	0.29
AutoCapture PCT 3-month (V)	560 (557)	0.66	0.25
AutoCapture PCT 6-month (V)	543 (538)	0.71	0.29
AutoCapture PCT 12-month (V)	447 (443)	0.77	0.29
AutoCapture PCT 24-month (V)	112 (111)	0.81	0.28
	**N (N > 5 mV)**	**Mean**	**Std. Dev**
R-wave 1-month (mV)	531 (508)	11.6	3.1
R-wave 3-month (mV)	531 (512)	11.8	3.1
R-wave 6-month (mV)	524 (505)	11.8	3.1
R-wave 12-month (mV)	434 (412)	11.7	3.2
R-wave 24-month (mV)	113 (105)	11.7	3.4

### AutoCapture activation and manual PCT comparison

AutoCapture setup was attempted and successfully activated in the majority of patients (619/644, 97%). Inability to activate in rare cases was primarily due to inadequate safety margins and atrial tachyarrhythmias causing fusion. No complications were observed related to the use and activation of AutoCapture algorithm during implant or remote follow-up.

**Fig. 1:**
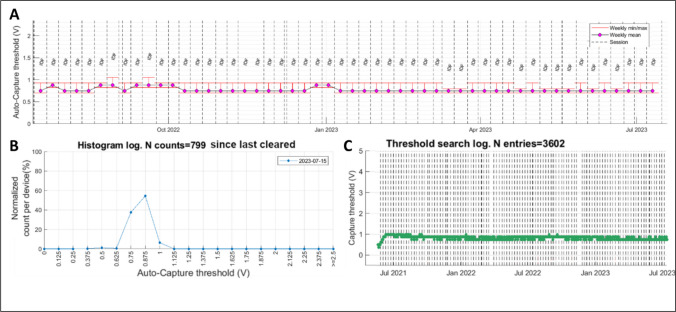
Example of AutoCapture pacing capture threshold diagnostics composed from all available remote data transmissions in a dual-chamber device over 2 years of follow-up. **A** weekly trend of mean, max and min automatically measured PCT showing stability over follow-up. Vertical dashed lines represent remote data transmission event. **B** Histogram of automatic PCT measurements performed showing consistent capture between 0.750 to 0.875 V. **C** AutoCapture measurements of PCT performed every 8 h since implant showing initial slight rise followed by stabilization over the following 2 years

Figure 1 shows an example of AutoCapture measured PCT data concatenated from remote device transmissions over 2-year follow up in a dual-chamber device.

**Fig. 2:**
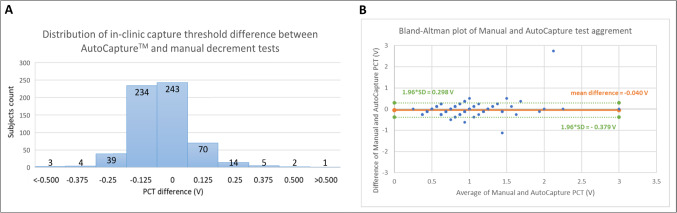
Comparison of in-clinic PCT manual decrement test and AutoCapture algorithm. A: Distribution and subject count of PCT difference shows 97.6% of devices had agreement of automated vs manual PCT within 0.25 V. B: Bland–Altman analysis shows excellent agreement with a mean difference of −0.040 V and 1.96 standard deviation of + 0.298 and −0.379 V between automated and manual PCT

Both AutoCapture and manual PCTs at implant were available in 615 patients and were within 0.25 V in 600 (97.6%) patients with average PCTs of 0.76 ± 0.28 and 0.80 ± 0.26 V, respectively, at a pulse width of 0.5 ms (Fig. 2A). Paired mean difference between PCT measured by both methods was small at −0.04 V difference [95%CI: −0.05 to −0.02] (paired t-test *p* < 0.001) with strong correlation between thresholds measured automatically by AutoCapture and manually (Pearson r = 0.794, *p* < 0.001). Bland–Altman analysis showed minimal bias and narrow error bounds (mean difference −0.040 V, 1.96 standard deviation −0.378 V and 0.298 V) shown in Fig. 2B.

### Follow-up electrical performance

At the most recent remote follow-up (median 521 [IQR: 347–693] days), 606/619 (97.9%) patients had AutoCapture enabled. Bipolar pacing (unipolar evoked response RV-tip to Case) was selected in 612/619 (99%) and the other 7 had unipolar pacing. One device had a change in pacing polarity to unipolar. Lead impedance measured by the device was 536 ± 60 ohms and sensed R-wave amplitude measured by the device was 11 ± 3 mV.

**Fig. 3:**
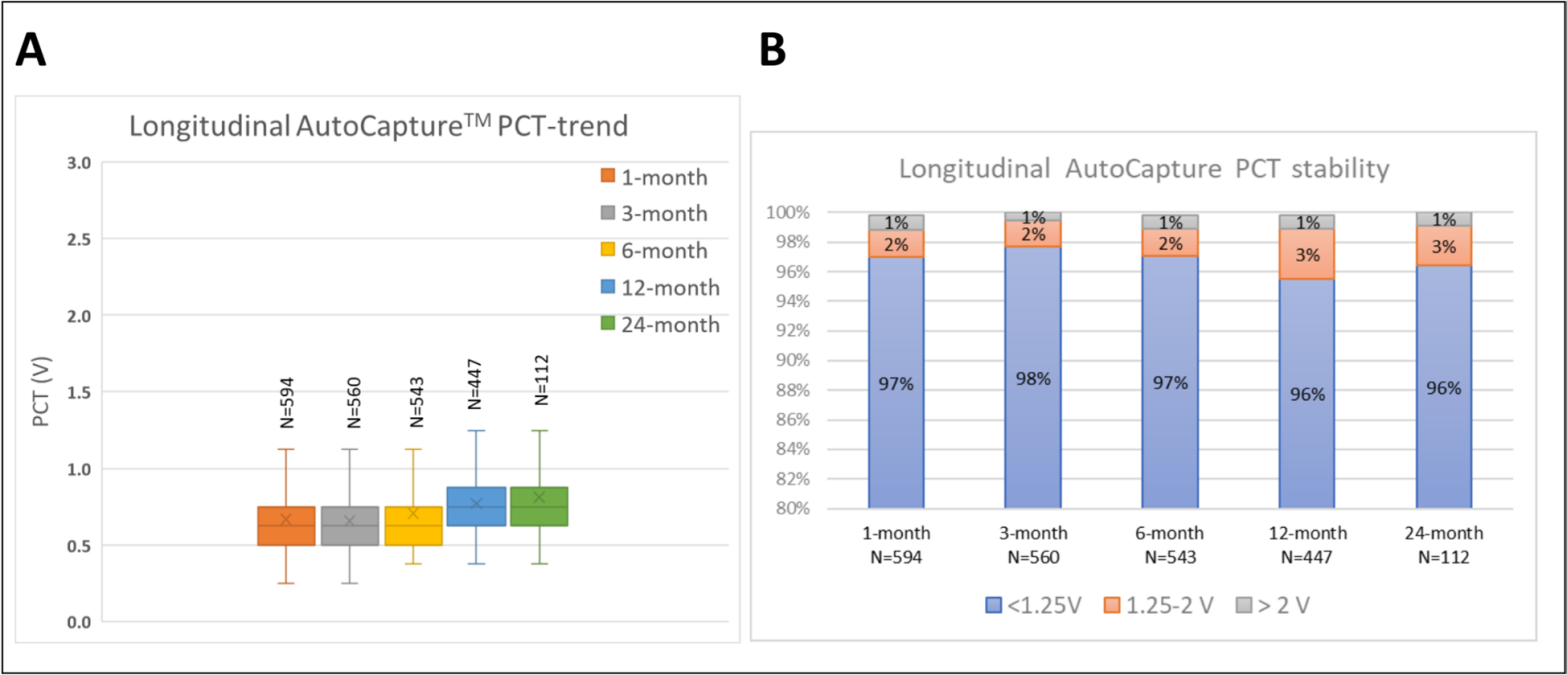
Longitudinal AutoCapture PCT measurements and stability. **A** Automatically measured PCT by AutoCapture algorithm shows consistently low capture output over follow-up period. Box plots show automated PCT mean (x), 5, 25, 50, 75 and 95 percentiles as well as number of devices at follow-up timepoints of 1-month, 3-month, 6-month, 12-month and 24-month. **B** Percentage and count of devices with non-elevated pacing capture output (< 1.25 V) remains consistently very high > 96% over all follow-up timepoints as compared to elevated (1.25–2 V and > 2 V) capture outputs

At 1-month, 3-month, 6-month, 12-month, and 24-month remote follow-up, average AutoCapture PCTs were 0.67 V ± 0.29 (*n* = 594), 0.66 V ± 0.25 (*n* = 560), 0.71 V ± 0.29 (*n* = 543), 0.77 V ± 0.29 (*n* = 447) and 0.81 V ± 0.28 (*n* = 112), respectively (Fig. 3A). The mean increase of AutoCapture measured PCT between 1-month and 12-month was 0.139 V ± 0.318 (*n* = 426). The percentage of patients with low (< 1.25 V) capture output remained stable over all follow-up time points (97%, 98%, 97%, 96%, 96%, respectively). All Chi-squared proportion comparisons were non-significant to baseline (*p* > 0.05, Fig. 3B).

Average sensed R-wave amplitude remained stable and measured 11.6 mV ± 3.1 (*n* = 531), 11.8 mV ± 3.1 (*n* = 531), 11.8 mV ± 3.1 (*n* = 524), 11.7 mV ± 3.2 (*n* = 434) and 11.7 mV ± 3.4 (*n* = 113), respectively over the same follow-up, with the percentage of subjects with > 5 mV remaining very high (96%, 96%, 96%, 95%, 93%, respectively). The 8 devices at 2-year time point with < 5 mV had persistently low R-wave amplitude (3.0–7.2 mV at 1-month and 2.8–4.8 mV at 2-year). Other device programming is described in Supplementary Section 3.0.

## Discussion

### Main findings

The main finding of our study is the medium-term consistency and reliability of the AutoCapture algorithm in a large cohort of patients receiving LBBAP therapy. The AutoCapture PCT measurement was consistently accurate when compared to the manually measured PCT. Over 2 years of remote follow-up, AutoCapture measured PCTs showed stability with only a minimal increase in the mean PCT during the follow-up period.

### Comparison of manual and automatic PCT measurement

In the comparison of manual thresholds with automatic thresholds, no statistically significant differences were observed and 97.6% of patients showed ≤ 0.25 V difference between the two methods.

A recent study of 45 patients receiving LBBAP during short-term 3-month follow-up has shown that automated PCT measurements are equivalent to manual testing in determining capture threshold [12]. Another study comparing performance of automatic capture confirmation algorithms across non-Abbott pulse generator manufactures showed similar automated PCT measurement accuracy [13]. Our study adds to this body of evidence and shows the accuracy and consistency of the AutoCapture algorithm in a large cohort of patients receiving LBBAP therapy paired with an Abbott pacemaker.

### AutoCapture algorithm feasibility in LBBAP

Myocardial injury and hyperpolarization after pacemaker lead fixation was shown to sometimes alter EVR leading to an acute increase of PCT or inability of ACC algorithms to activate and determine appropriate EVR sensitivity level [12]. In our study, it was possible to activate AutoCapture using the setup test in 622/644 or 97% of LBBAP patients and remained activated at a high rate (> 95%) similar to the Sola-Garcia et al. study which showed 96% to 100% success after chronic phase of first month [12]. AutoCapture setup test evaluates pacing at very low and very large pacing output in order to determine an appropriate safety margin over PCT to adequately distinguish loss of capture (LOC) or lead polarization artifacts from true tissue capture evoked response. The test subsequently configures EVR sensitivity level sufficiently higher than LOC and polarization. In instances of competitive rhythms and fusion, intrinsic events can interfere with setup test’s ability to safely recommend out-of-clinic beat-by-beat operation. Additionally out-of-clinic, AutoCapture’s inability to successfully obtain PCT due to fusion from competitive rhythm or lead instability can lead to initiating safety back-up pacing or high output pacing until PCT can be successfully measured by AutoCapture. Performing in-clinic setup test at a later time or adjusting device programming (such as AV delay, maximum tracking rate) can mitigate unsuccessful setup test and backup or high-output pacing.

In our cohort, bipolar pacing produced acceptable capture characteristics in 99% of patients. While the Sola-Garcia et al. study [12] had a lower proportion (50%) of the bipolar stimulation configuration, in the vast majority of our cases, the bipolar configuration yielded favorable ECG characteristics. Although unipolar stimulation configuration can provide lower PCT than bipolar pacing, lead impedance is about two-thirds of the bipolar lead impedance, which would negatively impact battery longevity. While AutoCapture supports capture confirmation with unipolar pacing and the few cases seemed to behave as expected, more investigation is needed to appropriately characterize behavior in this setting.

AutoCapture’s capability of performing beat-by-beat capture verification opens up possibility of delivering pacing at or just slightly above PCT which might lead to higher likelihood of selective left-bundle capture in cases where lead tip is appropriately positioned in the septum. Although labelling of capture type at implant and over time was not available in our study, such data would be of value to elucidate feasibility of obtaining long-term selective capture as well as any clinical benefit of selective versus other types of conduction system capture. Su et al. [14] found in a large single-center study that conduction system capture was lost at 1 year follow-up in only 2 out of 618 patients that had successful LBBP capture at implant. Taken together with our results, it appears from an algorithm performance perspective that AutoCapture’s ability to measure and maintain PCT stability over the 2-year follow-up period was unaffected by any potential variability in capture types.

### Medium-term trend of AutoCapture algorithm measurements

The stable trend of PCT determined by AutoCapture (with slight increase in the mean PCT of 0.1 V per year) compares favorably with manually measured PCT in a meta-analysis from 45 centers with 6061 patients receiving lumen-less leads by Ellenbogen et al. [15] who reported average measured PCT of 0.66 V at 1-month and 0.75 V at 18-month follow-up. R-wave amplitude in this study also compares well with our results showing an average of 13.8 mV at 1-month and 13.9 mV at 18-month. Battery longevity improvement incurred due to AutoCapture narrow safety pacing margin during beat-by-beat confirmation operation is variable and depends on multiple factors including base pacing rate, atrial and ventricular pacing burden, lead impedances, pacing safety margin, device algorithms controlling AV delay and PCT. The largest battery saving would be seen with elevated PCT (> 1.5 V) where using larger (e.g. 1.5x) safety margin would cause pacing voltage doubler activation.

### Study limitations

This is a single-center, observational, non-randomized, retrospective study and results should be evaluated with all limitations inherent to such study design. However, the large sample size and consistency of the gathered data we believe allows for relatively reliable observations with regards to algorithm performance trends. In addition, our center’s extensive experience over several years with LBBAP implants and subsequent follow-up and management may have impacted or improved the relative consistency of these study results and may be difficult to extend or extrapolate to centers that are newer to, or just beginning a LBBAP program. The study didn’t analyze any correlation with clinical endpoints such as hospitalization, symptoms, lead revision, or device-related complications. Also, procedural characteristics (including capture type and anodal capture) and clinical follow-up information was not systematically collected and was not available. Such data would provide better interpretation of the real-world significance of the study results. The study included a much larger percentage of dual-chamber with bipolar pacing and results need to be verified with larger datasets that includes single-chamber or unipolar pacing.

## Conclusion

The AutoCapture™ algorithm measured accurate PCTs compared to manual in-clinic measurements and showed a stable trend during medium-term follow-up in pacemaker patients receiving LBBAP. No clinical complications were related to AutoCapture activation or use. Expanding the use of the algorithm to patients with LBBAP could extend its benefits of improved device longevity, beat-by-beat capture confirmation, and remote monitoring of capture parameters, thereby further enhancing patient safety. The ability to pace and capture at lower outputs may enable clinicians to selectively favor left bundle branch mediated ventricular conduction.

## Supplementary Information

Below is the link to the electronic supplementary material.

Supplementary file1 (DOCX 18 KB)
