# Supplementary material for: Performance of automatic capture confirmation algorithm in a large cohort of pacemaker patients with left bundle branch area pacing
Source: J Interv Card Electrophysiol. 2026 Feb 23;69(4):729–35. doi: 10.1007/s10840-025-02229-y (PMC13221406; doi:10.1007/s10840-025-02229-y)
Supplement: Supplementary file 1 — Supplementary file1 (DOCX 18 KB) [file 10840_2025_2229_MOESM1_ESM.docx]

# Supplementary Material

**1.0 Characteristics of subgroup of excluded devices**

Table A1 shows characteristics of 25 excluded device data (3 had < 30 days follow-up, 20 didn’t have AutoCapture setup test performed, 2 had setup test didn’t pass).

Table A1 – Characteristics of subgroup of excluded devices

|  | Excluded | | | |
| --- | --- | --- | --- | --- |
|  | **N** | **%** | **mean** | **std** |
| Device count | 25 | 100% | na | na |
| Patient age (year) | 23 | 92% | 82 | 6 |
| Follow-up (day) | 23 | 92% | 470 | 265 |
| Dual-chamber Pacer | 21 | 84% | na | na |
| Tendril 2088 lead | 0 | 0% | na | na |
| SelectSecure 3830 lead | 25 | 100% | na | na |
| Sensed AVD (ms) | 23 | 92% | 148 | 33 |
| Paced AVD (ms) | 23 | 92% | 178 | 32 |
| RA pacing burden (%) | 21 | 84% | 22 | 35 |
| RV pacing burden (%) | 25 | 100% | 74 | 37 |
| Bipolar pacing configuration | 24 | 96% | na | na |
| Lead impedance most recent (ohm) | 24 | 96% | 494 | 122 |
| R-wave most recent (mV) | 18 | 72% | 10.4 | 3.5 |
| Automatic AutoCapture PCT (V) | na | na | na | na |
| Manual PCT (V) | 25 | 100% | 0.49 | 0.13 |

**2.0 Characteristics of subgroups lumenless and SDL leads**

Table A2 below shows comparison of characteristics in subgroup of patients who received Abbott Tendril 2088 stylet-driven lead (SDL) and subgroup who received SelectSecure 3830 lumenless lead. The sample size of Tendril subgroup is very small owning to wider early adoption and experience of the lumenless lead in the timeline of the study data while stylet-driven leads experience came in later and had much shorter follow-up time. Characteristics and implant techniques of stylet-driven leads are currently much more understood and approved by regulatory agencies for delivery LBBAP pacing therapy.

Table A2. Characteristics of Tendril stylet-driven and SelectSecure lumenless leads subgroups

|  | Tendril 2088 | | | | SelectSecure 3830 | | | |
| --- | --- | --- | --- | --- | --- | --- | --- | --- |
|  | **N** | **%** | **mean** | **std** | **N** | **%** | **mean** | **std** |
| Device count | 14 | 2% | na | na | 630 | 98% | na | na |
| Patient age | 13 | 93% | 81 | 8 | 617 | 98% | 80 | 9 |
| Follow-up days | 14 | 100% | 71 | 32 | 630 | 100% | 531 | 258 |
| Dual-chamber Pacer | 12 | 86% | na | na | 519 | 82% | na | na |
| Included in PCT comparison | 14 | 100% | na | na | 606 | 96% | na | na |
| Sensed AVD (ms) | 12 | 86% | 138 | 37 | 519 | 82% | 152 | 20 |
| Paced AVD (ms) | 12 | 86% | 166 | 47 | 519 | 82% | 187 | 18 |
| RA pacing burden (%) | 12 | 86% | 40 | 37 | 519 | 82% | 31 | 34 |
| RV pacing burden (%) | 14 | 86% | 72 | 43 | 623 | 99% | 75 | 38 |
| Bipolar pacing configuration | 14 | 100% | na | na | 603 | 96% | na | na |
| Lead impedance most recent (ohm) | 14 | 100% | 445 | 54 | 623 | 99% | 532 | 65 |
| R-wave most recent (mV) | 14 | 93% | 10.6 | 3.4 | 606 | 96% | 11.7 | 3.2 |
| Automatic AutoCapture PCT (V) | 13 | 93% | 0.55 | 0.14 | 606 | 96% | 0.76 | 0.28 |
| Manual PCT (V) | 13 | 93% | 0.60 | 0.13 | 606 | 96% | 0.80 | 0.25 |
| AutoCapture Manual PCT delta (V) | 13 | 93% | -0.05 | 0.11 | 606 | 96% | -0.04 | 0.17 |
| AutoCapture PCT 1-month (V) | 11 | 79% | 0.65 | 0.09 | 562 | 89% | 0.66 | 0.25 |
| AutoCapture PCT 3-month (V) | 3 | 21% | 0.63 | 0.00 | 559 | 89% | 0.66 | 0.25 |
| AutoCapture PCT 6-month (V) | 0 | 0% | na | na | 543 | 86% | 0.71 | 0.29 |
| AutoCapture PCT 12-month (V) | 0 | 0% | na | na | 448 | 71% | 0.77 | 0.29 |
| AutoCapture PCT 24-month (V) | 0 | 0% | na | na | 113 | 18% | 0.81 | 0.28 |

**3.0 AutoCapture setup test and other device programming information**

AutoCapture PCT was compared to Manual PCT recorded during in-clinic session. AutoCapture setup test was configured with base pacing rate faster than intrinsic and AV delay (programmable between sensed/paced: 25/50, 70/100, 100/130) to avoid competitive rhythm or ventricular fusion. Bipolar polarity was attempted as first option and unipolar attempted as second option. AutoCapture nominal parameters are base pacing rate of 60 bpm, starting threshold 1.5 V, sensed/paced AV delay during test 25/50 ms, pacing pulse width 0.4 ms and backup pacing channel bipolar tip-to-ring. EGM waveforms are automatically recorded during AutoCapture setup and ventricular capture test and used for retrospective data analysis.
